# A modified Sequential Organ Failure Assessment score for dengue: development, evaluation and proposal for use in clinical trials

**DOI:** 10.1186/s12879-022-07705-8

**Published:** 2022-09-03

**Authors:** Angela McBride, Nguyen Lam Vuong, Nguyen Van Hao, Nguyen Quang Huy, Ho Quang Chanh, Nguyen Thi Xuan Chau, Nguyen Minh Nguyet, Damien K. Ming, Nguyen Thanh Ngoc, Phung Tran Huy Nhat, Nguyen Thanh Phong, Luong Thi Hue Tai, Phan Vinh Tho, Dinh The Trung, Dong Thi Hoai Tam, Huynh Trung Trieu, Ronald Bertus Geskus, Martin J. Llewelyn, C. Louise Thwaites, Sophie Yacoub

**Affiliations:** 1 Oxford University Clinical Research Unit, Ho Chi Minh City, Vietnam; 2 Department of Global Health and Infection, Brighton and Sussex Medical School, Brighton, UK; 3 University of Medicine and Pharmacy at Ho Chi Minh City, Ho Chi Minh City, Vietnam; 4 Hospital for Tropical Disease, Ho Chi Minh City, Vietnam; 5 Centre for Tropical Medicine and Global Health, University of Oxford, Oxford, UK; 6 Department of Infectious Disease, Imperial College London, London, UK

**Keywords:** Dengue, Shock, SOFA, Modified SOFA, Delta SOFA, Clinical trial endpoint, Vietnam

## Abstract

**Background:**

Dengue is a neglected tropical disease, for which no therapeutic agents have shown clinical efficacy to date. Clinical trials have used strikingly variable clinical endpoints, which hampers reproducibility and comparability of findings. We investigated a delta modified Sequential Organ Failure Assessment (delta mSOFA) score as a uniform composite clinical endpoint for use in clinical trials investigating therapeutics for moderate and severe dengue.

**Methods:**

We developed a modified SOFA score for dengue, measured and evaluated its performance at baseline and 48 h after enrolment in a prospective observational cohort of 124 adults admitted to a tertiary referral hospital in Vietnam with dengue shock. The modified SOFA score included pulse pressure in the cardiovascular component. Binary logistic regression, cox proportional hazard and linear regression models were used to estimate association between mSOFA, delta mSOFA and clinical outcomes.

**Results:**

The analysis included 124 adults with dengue shock. 29 (23.4%) patients required ICU admission for organ support or due to persistent haemodynamic instability: 9/124 (7.3%) required mechanical ventilation, 8/124 (6.5%) required vasopressors, 6/124 (4.8%) required haemofiltration and 5/124 (4.0%) patients died. In univariate analyses, higher baseline and delta (48 h) mSOFA score for dengue were associated with admission to ICU, requirement for organ support and mortality, duration of ICU and hospital admission and IV fluid use.

**Conclusions:**

The baseline and delta mSOFA scores for dengue performed well to discriminate patients with dengue shock by clinical outcomes, including duration of ICU and hospital admission, requirement for organ support and death. We plan to use delta mSOFA as the primary endpoint in an upcoming host-directed therapeutic trial and investigate the performance of this score in other phenotypes of severe dengue in adults and children.

**Supplementary Information:**

The online version contains supplementary material available at 10.1186/s12879-022-07705-8.

## Background

Dengue places huge burdens on the healthcare services and economies of dengue-endemic regions, where approximately 50% of the world’s population live [1]. Complex interactions between host and viral factors make the clinical presentation highly variable. Although the majority of dengue virus (DENV) infections are asymptomatic or self-limiting, a small proportion of symptomatic patients develop features of severe disease, which include severe plasma leakage with hypovolaemic shock, significant bleeding and/or major organ impairment.

At present, guidelines for dengue treatment are limited to supportive care and careful fluid management [2]; clinical trials investigating antivirals and immune modulators have been conducted, but none have demonstrated efficacy by virological or clinical endpoints [3–6]. The clinical endpoints used in dengue research are strikingly variable, hampering reproducibility and comparability of findings. Seeking to correct this, an expert working group have recommended endpoints for use in early phase trials [7, 8], including definitions for moderate and severe plasma leakage, bleeding, and organ involvement. Pending validation, the endpoints proposed by the above studies may be applicable to interventional studies involving treatment of patients before the onset of severe dengue (e.g., antiviral candidates in early infection, host-directed immune modulation in high-risk groups). However, patients presenting with established dengue shock already fulfil these categorical endpoints at baseline. Thus, more sensitive and dynamic markers of clinical progress with capacity to reflect both improvement and worsening over time are needed for patients with dengue shock. Further, Dengue is a multisystem disease and splitting endpoints into categories including vascular leakage, severe bleeding and organ impairment may fail to capture the whole clinical picture. As such, we believe that there is a role for using composite endpoints to assess dengue severity as a single entity, rather than scoring separate syndromes.

One possibility is the extension and/or modification of the Sequential Organ Failure Assessment (SOFA) score for dengue. The SOFA score was originally developed to quantify organ impairment in sepsis and takes into account functional impairment of six end-organ systems (respiratory, coagulation, cardiovascular, hepatic, renal and neurological) [9]. The simpler quick SOFA (qSOFA) is a good screening tool for bacterial sepsis, and it has been found to perform well as predictor for mortality in non-bacterial causes of sepsis in low middle income countries, including dengue [10]. However, qSOFA was not intended for use beyond triage, and is insufficiently sensitive to monitor clinical progress in patients with established sepsis. Recently, there has been growing consensus for the use of the full SOFA score and its derivatives as clinical endpoints in randomized controlled trials based in the intensive care unit (ICU) [9, 11]. However, modification of the full SOFA score for dengue might be required; dengue has some unique pathophysiological phenomena including narrowed pulse pressure, which should be considered for inclusion in the cardiovascular component. Narrowing of the pulse pressure is one of the earliest manifestations of dengue shock and occurs prior to the development of hypotension [2]. The pulse pressure is also used as a resuscitation target for fluid management and a prognostic factor in dengue shock [12, 13]. In addition, arterial puncture for PaO_2_ measurement is relatively contraindicated in thrombocytopaenia, and frequently unavailable in many dengue endemic settings.

We therefore set out to develop a modified SOFA score for dengue, determine how it performed to predict clinical outcomes in adults with dengue shock, to understand what relative contribution the modified and original SOFA parameters conferred on the prognostic ability of the score, and whether a change in SOFA score (delta SOFA) over the first 48 h of treatment might be a feasible composite endpoint for therapeutic trials in severe dengue.

## Methods

### Clinical study

This prospective observational cohort study was conducted at the Hospital for Tropical Diseases (HTD), a tertiary referral center for infectious diseases in Ho Chi Minh City, Vietnam. Patients were recruited between June 2019 and June 2021. Ethical approval was obtained from the local ethics committee at HTD and Oxford Tropical Research Ethics Committee.

Patients ≥ 16 years of age were recruited following admission to either the emergency department (ED) or adult ICU, with a clinical diagnosis of dengue shock in line with the 2009 World Health Organization (WHO) criteria [2]. Patients were excluded from the study if they had been unwell for ≥ 7 days at presentation or had been treated for shock for ≥ 24 h in any healthcare setting prior to recruitment. Written informed consent was obtained from patients, or their relatives if the patient was unable to consent due to severity of illness. Participants < 18 years of age provided written assent in addition to written parental/guardian consent.

Clinical assessments and laboratory parameters necessary to compute the modified SOFA score for dengue (Table 1) were collected at enrolment and 48 h later, except for bilirubin which was only measured at baseline. Blood pressure was monitored hourly during the fluid resuscitation and subsequently as clinically indicated, using either a manual sphygmomanometer or invasive blood pressure monitoring. Pulse pressure was calculated by subtracting the diastolic from systolic blood pressure value at the time of assessment.

**Table 1:** Modified SOFA score for dengue

System	Score				
	0	1	2	3	4
Respiratory system^(*)^					
SpO_2_/FiO_2_, mmHg	≥ 400	< 400	< 315	< 235 and/or respiratory support	< 150 with respiratory support
Coagulation					
Platelets, /μL	≥ 150,000	< 150,000	< 100,000	< 50,000	< 20,000
Liver function					
Bilirubin, mg/dl [μmol/l]	< 1.2 [20]	1.2–1.9 [20–32]	2.0–5.9 [33–101]	6.0–11.9 [102–204]	> 12.0 [204]
Cardiovascular system					
MAP/**PP** and adrenergic agents	MAP ≥ 70 mmHg & **PP ≥ 20 mmHg**	MAP < 70 mmHg or **PP < 20 mmHg**	**PP < 10 mmHg** OR dopamine ≤ 5 μg/kg/min or dobutamin (any dose)	Dopamine 5.1-15 μg/kg/min OR epinephrine ≤ 0.1 μg/kg/min OR norepinephrine ≤ 0.1 μg/kg/min	Dopamine > 15 μg/kg/min OR epinephrine > 0.1 μg/kg/min OR norephinephrine > 0.1 μg/kg/min
Central nervous system					
Glasgow Coma Scale score	15	13–14	10–12	6–9	< 6
Renal function					
Creatinine, mg/dL	< 1.2	1.2–1.9	2.0–3.4	3.5–4.9	> 5.0
Urine output, mL/d				< 500	< 200

Bold type indicates a modification of the cardiovascular SOFA parameter to include pulse pressure (PP) (as per WHO dengue shock classification 2009)

*FiO*_2_ fraction of inspired oxygen; *MAP* mean arterial pressure; *SpO*_2_ oxygen saturations

(*)This component is adpated form the work of Grissom et al. [14]

Clinical outcomes were: duration of ICU admission, requirement for organ support (mechanical ventilation, vasopressors, renal replacement therapy), duration of intravenous (IV) fluid therapy and death.

### Development of the modified SOFA score for dengue

The components of the modified SOFA score for dengue were selected to take into account particular features relevant to dengue, including addition of pulse pressure to the cardiovascular score to reflect the unique tendency of patients with dengue shock to maintain systolic blood pressure in the face of rising diastolic blood pressure as a compensatory mechanism during profound plasma leakage.

We also use the SpO_2_/FiO_2_ ratio as a substitute for PaO_2_/FiO_2_ ratio due to the relative contraindication of arterial puncture in patients with severe thrombocytopaenia, and the fact that arterial blood gas analysis for PaO_2_ is not readily available as standard of care across the region [14].

The components of the modified SOFA score for dengue are highlighted in Table 1 (bold type). Where two parameters contribute to an organ specific failure score, the parameter scoring highest was chosen.

### Statistical methods

The mSOFA score for dengue has six components, each scored from 0 (the mildest) to 4 (the most severe). As bilirubin was only measured at baseline, the liver function component was not available on day 2. We therefore excluded the liver function component when calculating the delta mSOFA score for dengue, which was calculated by subtracting the total score for the five other components on day 0 from that on day 2.

For binary endpoints including ICU admission, mechanical ventilation, vasopressor support, haemofiltration, and mortality, a binary logistic regression model was used and reported by odds ratio (OR) with 95% confidence interval (CI) and prediction of the probability of having the endpoint. To evaluate the prognostic performance, Brier score rescaled to range from 0 to 1 (higher values indicating better performance) and area under the curve (AUC) were reported. Scaled Brier score was calculated by [1 − Brier score/Brier score of the null model]. Bootstrap procedure with 500 resamples with replacement was used to calculate 95% CI of these metrics. Firth’s bias correction was used in case there was clear separation of the score (or delta score) between the groups with and without a particular endpoint [15, 16]. For time-to-event endpoints including ICU discharge, hospital discharge, and stopping IV fluid use, a Cox proportional hazard model was used to estimate hazard ratio (HR) and 95% CI. For total volume of IV fluid use, a linear regression model with log-transformation of the endpoint was used to estimate mean ratio and 95% CI. Because death was a competing risk in time-to-event endpoints and total volume of IV fluid use, in all fatal cases the time-to-event was set to the highest value recorded for survivors plus one day, and the total volume of fluid received was set to the highest value recorded for survivors plus 100 ml. The baseline score and delta score were used as the only predictor with a linear effect in each model. Subgroup analyses were carried out for patients with and without comorbidities, and patients transferred from other hospitals. All analyses were done using the statistical software R version 4.1.0 [17].

## Results

### Demographics and clinical presentation

This analysis included 124 patients with dengue shock [50.8% males, median age 24.5 (25th; 75th percentiles: 20–32) years] (Table 2). Nine (7.4%) patients had known comorbidities at the time of admission to hospital, including: diabetes, hypertension, peptic ulcer disease, and moderate/severe liver or kidney disease. A majority of patients (59.7%) were diagnosed with dengue shock at another hospital; the remainder presented directly to HTD with established dengue shock. The median illness day at presentation was 5 (range 3–7). Twenty-nine patients (23.4%) were admitted for management in the ICU due to requirement for organ support or persistent haemodynamic instability after initial resuscitation; the remainder were stabilized in the ED for up to 48 h and transferred to a less acute ward. The median duration of hospital admission was 5 days. The median duration of ICU admission was 3 days. A minority of patients required organ support in ICU: mechanical ventilation (9/124, 7.3%), vasopressors (8/124, 6.5%), haemofiltration (6/124, 4.8%), and 5/124 (4.0%) patients died. Patients with comorbidities were more likely to be older, admitted to ICU, to require mechanical ventilation, and recieve platelet transfusion (Additional file 1: Table S1). There was no significant difference between the patients transferred from other hospitals and those who were admitted directly to HTD in terms of clinical data (Additional file 1: Table S2).

**Table 2:** Clinical data

	N	All patients Median (Q1; Q3) or n (%)
Age (years)	124	24.5 (20; 32)
Sex male	124	63 (50.8)
Comorbidities	124	9 (7.3)
Transferred from other hospital	124	74 (59.7)
BMI (kg/m^2^)	124	23.1 (19.9; 26.3)
Day of illness at time of shock onset	124	5 (4; 5)
ICU admission	124	29 (23.4)
ICU length of stay (days)	29	3 (2; 5)
Hospital length of stay (days)	124	5 (4; 7)
Mechanical ventilation	124	9 (7.3)
Length of mechanical ventilation support (days)	9	7 (4; 8)
Vasopressor support	124	8 (6.5)
Length of vasopressor support (days)	8	3 (1.8; 4.8)
Length of IV fluid use (days)	124	2 (2; 2)
Total volume of IV fluid use (L)	124	4.7 (3.5; 6.3)
Haemofiltration	124	6 (4.8)
Length of haemofiltration use (days)	6	5.5 (3.2; 7.0)
Death	124	5 (4.0)

*BMI* body mass index; *ICU* intensive care unit; *IV* intravenous; *Q1* first quartile (25th percentile); *Q3* third quartile (75th percentile)

### Association between baseline mSOFA score for dengue and clinical outcomes

**Fig. 1:**
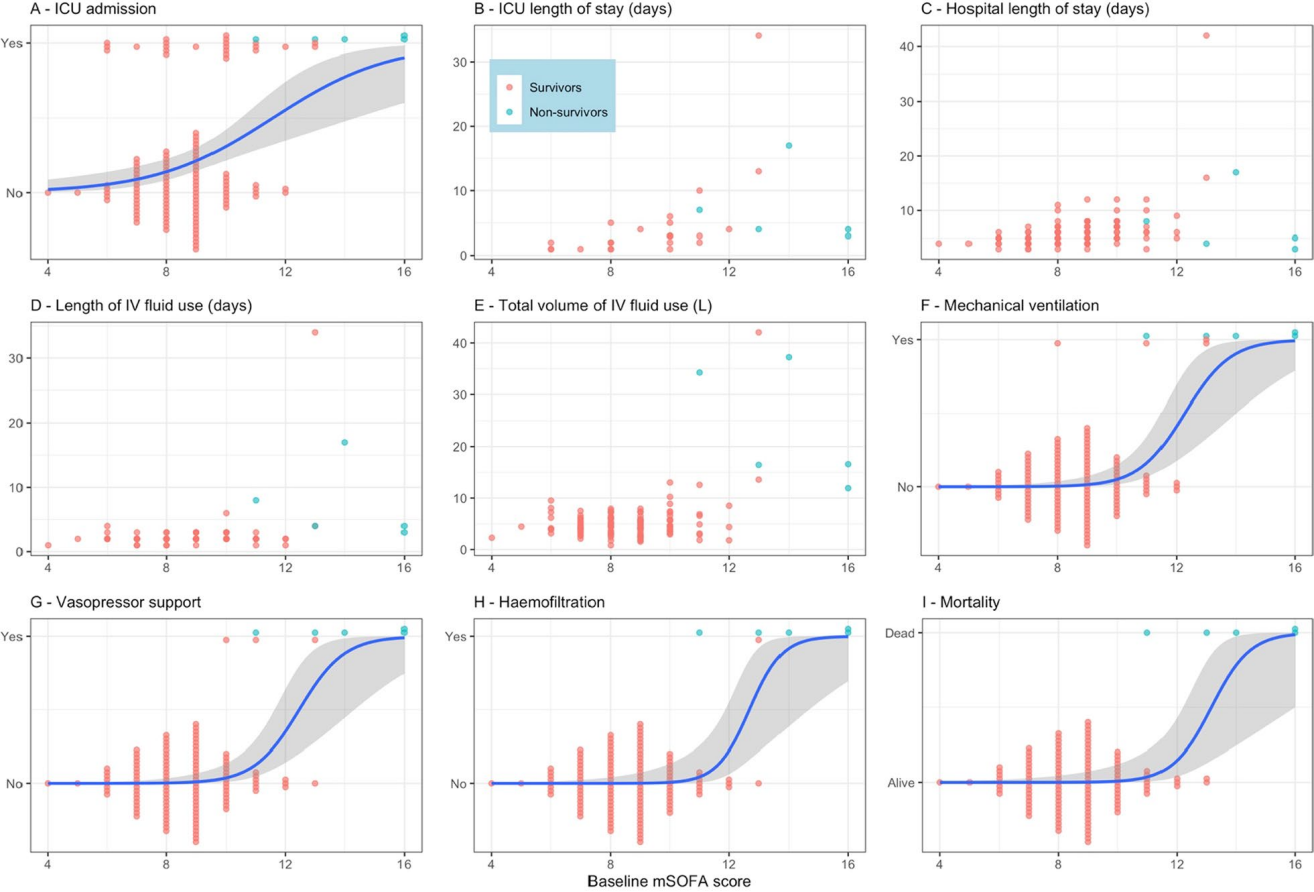
Association between baseline mSOFA score and endpoints. In **A**, **F**, **G**, **H** and **I** the probability of having the endpoint is estimated from the binary logistic regression model with a predictor baseline mSOFA score; the blue curve represents the estimated probability and the gray region represents 95% confidence interval. In all plots (**A**–**I**), each point represents an individual measurement and is coloured by survivor (red) and non-survivor (blue)

The median (25th; 75th percentiles) baseline mSOFA score for dengue was 9 (8; 10). In univariate analyses, higher baseline mSOFA score for dengue was associated with the following clinical endpoints: admission to ICU (OR 1.66, 95% CI 1.30–2.23 for each 1-point increase of baseline mSOFA score), mechanical ventilation (OR 3.66, 95% CI 2.09–8.49), vasopressor support (OR 3.73, 95% CI 2.08–9.17), haemofiltration (OR 6.24, 95% CI 2.48–39.9) and mortality (OR 4.60, 95% CI 2.12–22.8) (Table 3, Fig. 1). Higher mSOFA score for dengue was associated with increased: time to ICU discharge (HR 0.64, 95% CI 0.52–0.78), time to hospital discharge (HR 0.66, 95% CI 0.59–0.75), time to stopping IV fluid therapy (HR 0.82, 95% CI 0.75–0.90), and total volume of IV fluid use (mean ratio 1.18, 95% CI 1.12–1.25) (Table 3, Fig. 1). mSOFA was more strongly associated with clinical outcomes than baseline pulse pressure alone (Table 3, Additional file 1: Table S6).

**Table 3:** Results from models with baseline mSOFA and delta mSOFA score

	Est	Baseline mSOFA score				Delta mSOFA score			
		Est (95% CI)	p	Scaled Brier score (95% CI)	AUC (95% CI)	Est (95% CI)	p	Scaled Brier score (95% CI)	AUC (95% CI)
ICU admission	OR	1.66 (1.30; 2.23)	< 0.001	0.20 (0.06; 0.39)	0.71 (0.59; 0.83)	1.55 (1.21; 2.10)	0.002	0.16 (0.03; 0.31)	0.66 (0.53; 0.78)
ICU discharge	HR	0.64 (0.52; 0.78)	< 0.001	–	–	0.68 (0.56; 0.84)	< 0.001	–	–
Hospital discharge	HR	0.66 (0.59; 0.75)	< 0.001	–	–	0.84 (0.77; 0.91)	< 0.001	–	–
Stop IV fluid use	HR	0.82 (0.75; 0.90)	< 0.001	–	–	0.76 (0.69; 0.84)	< 0.001	–	–
Total volume of IV fluid use	MR	1.18 (1.12; 1.25)	< 0.001	–	–	1.19 (1.14; 1.25)	< 0.001	–	–
Mechanical ventilation	OR	3.66 (2.09; 8.49)	< 0.001	0.56 (0.29; 0.97)	0.92 (0.75; 1.00)	3.64 (1.92; 9.84)	0.002	0.52 (0.23; 0.91)	0.93 (0.81; 1.00)
Vasopressor support	OR	3.73 (2.08; 9.17)	< 0.001	0.51 (0.18; 0.86)	0.96 (0.91; 1.00)	1.95 (1.40; 3.16)	< 0.001	0.45 (0.02; 0.97)	0.76 (0.47; 1.00)
Haemofiltration	OR	6.24 (2.48; 39.9)	0.005	0.78 (0.33; 0.98)	0.99 (0.96; 1.00)	4.90 (1.98; 137)	< 0.001	0.75 (0.63; 0.99)	1.00 (0.99; 1.00)
Mortality	OR	4.60 (2.12; 22.8)	0.006	0.74 (0.19; 0.97)	0.98 (0.95; 1.00)	3.75 (1.86; 38.4)	< 0.001	1.00 (0.88; 1.00)	1.00 (1.00; 1.00)

All parameters are estimated from models with only one predictor ‘baseline mSOFA score’ or ‘delta mSOFA score’ with a linear effect. Logistic regression model is used for binary endpoints (ICU admission, mechanical ventilation, vasopressor support, haemofiltration, and death), Cox proportional hazard model is used for time-to-event endpoints (ICU discharge, hospital discharge, and stop IV fluid use), and linear regression model is used for total volume of IV fluid use with log-transformation. The estimates (OR, HR, or MR) and 95% CIs are reported for each point increase of baseline mSOFA or delta mSOFA score

*AUC* area under the curve; *CI* confidence interval; *Est* estimate; *HR* hazard ratio; *ICU* intensive care unit; *IV* intravenous fluid; *MR* mean ratio; *OR* odds ratio

### Association between delta mSOFA for dengue and clinical outcomes

**Fig. 2:**
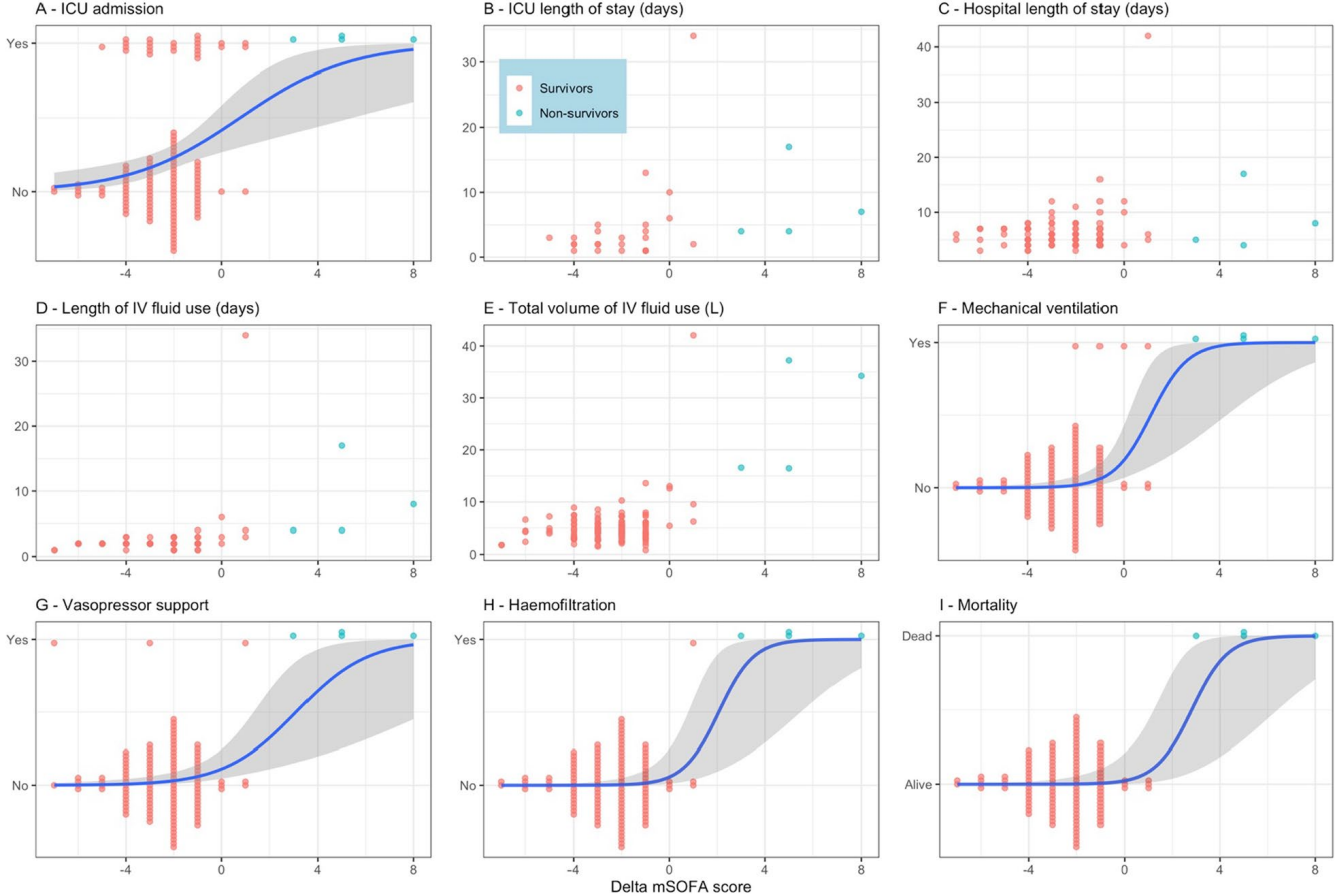
Association between delta mSOFA score and endpoints. In **A**, **F**, **G**, **H** and **I** the probability of having the endpoint is estimated from the binary logistic regression model with a predictor delta mSOFA score; the blue curve represents the estimated probability and the gray region represents 95% confidence interval. In all plots (**A**–**I**), each point represents an individual measurement and is coloured by survivor (red) and non-survivor (blue)

The median (25th; 75th percentiles) mSOFA score for dengue (minus bilirubin) decreased from 6 (5; 6) at baseline to 3 (2; 4) at 48 h. Median (25th; 75th percentiles) delta mSOFA score was − 2 (− 3; − 1). Higher delta mSOFA for dengue (i.e. less improvement over 48 h) was associated with admission to ICU (OR 1.55, 95% CI 1.21–2.10 for each 1-point increase of delta mSOFA), requirement for mechanical ventilation (OR 3.64, 95% CI 1.92–9.84), vasopressors (OR 1.95, 95% CI 1.40–3.16) and haemofiltration (OR 4.90, 95% CI 1.98–137), and mortality (OR 3.75, 95% CI 1.86–38.4) (Table 3, Fig. 2). Higher delta mSOFA score for dengue was associated with longer time to ICU discharge (HR 0.68, 95% CI 0.56–0.84), time to hospital discharge (HR 0.84, 95% CI 0.77–0.91), time to stopping IV fluid therapy (HR 0.76, 95% 0.69–0.84), and total volume of IV fluid use (mean ratio 1.19, 95% CI 1.14–1.25) (Table 3, Fig. 2).

### Contribution of individual components to baseline mSOFA and delta mSOFA score for dengue

**Fig. 3:**
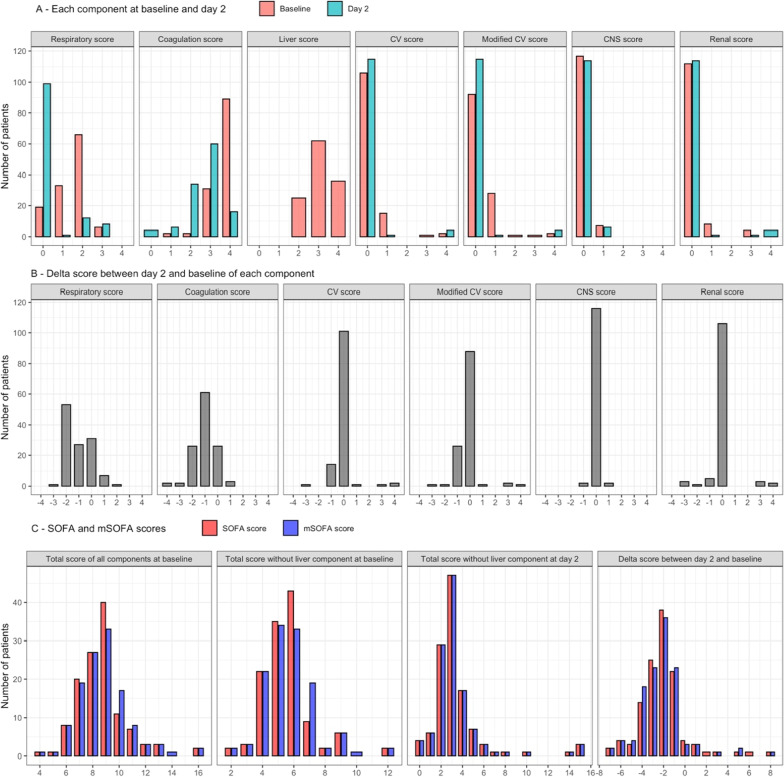
**A** Distribution of each component of mSOFA score at baseline (red) and day 2 (blue), **B** Distribution of each component of delta mSOFA score between day 2 and baseline, **C** Distribution of total mSOFA score (blue) and original SOFA score (red) in all patients

Considering the breakdown of the mSOFA score for dengue, most patients had elevated scores for the respiratory, coagulation, and hepatic components. Few patients had high scores for the cardiovascular, central nervous system and renal components (Fig. 3A, B). The cardiovascular component in the mSOFA for dengue was higher than the original SOFA score, due to the addition of pulse pressure, which led to an increase of 1 point compared to the original SOFA score in 15 patients at baseline (Fig. 3C). However, these scores were equal in all patients at 48 h.

**Fig. 4:**
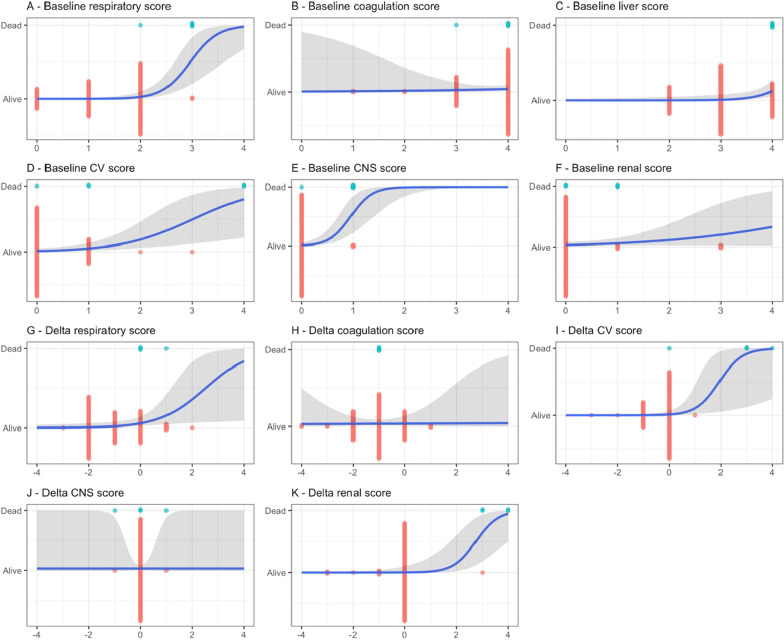
**A-K** show the association between each component of mSOFA score and mortality. The probability of having the endpoint is estimated from the binary logistic regression model with a predictor delta mSOFA score; in all panels the blue curve represents the estimated probability and the gray region represents 95% confidence interval. Each point represents an individual measurement and is coloured by survivor (red) and non-survivor (blue). *CNS* central nervous system; *CV* cardiovascular

There was association between individual components of the mSOFA for dengue, measured at baseline and mortality: respiratory, cardiovascular, central nervous system and hepatic components were strongly associated; renal score was associated but less strongly than the others. However, baseline coagulation score was not associated with mortality (Table 4, Fig. 4A–F). With respect to delta individual components of mSOFA for dengue, delta respiratory score, delta cardiovascular score and delta renal score were associated with mortality. There was no association between delta mSOFA coagulation and/or central nervous system scores and death (Table 4, Fig. 4G–K).

**Table 4:** Association between each component of mSOFA score and death

Component	Baseline individual mSOFA			Delta individual mSOFA		
	OR	95% CI	p	OR	95% CI	p
Respiratory system	78.22	10.28; 1075	< 0.001	3.02	1.24; 9.38	0.014
Coagulation	1.06	0.33; 9.99	0.940	0.98	0.35; 3.35	0.973
Cardiovascular system	3.74	1.89; 9.68	< 0.001	6.75	2.75; 164	< 0.001
Central nervous system	99.86	14.08; 1236	< 0.001	1.00	0.01; 79.6	1
Renal function	2.11	0.87; 4.30	0.088	7.36	3.10; 49.9	< 0.001
Liver function	20.90	2.51; 2740	0.002	–	–	–

ORs and 95% CIs were calculated from binary logistic regression model with Firth’s bias correction for each point increase

*OR* odds ratio; *CI* confidence interval

### Comparison of original SOFA and mSOFA for dengue

We compared how the mSOFA for dengue performed relative to the original SOFA score (though we were unable to investigate the performance of SpO_2_ relative to PaO_2_ in the respiratory component, as this was not available). At baseline, both scores performed similarly well to predict clinical endpoints. Delta mSOFA for dengue and delta SOFA also performed very similarly in predicting need for organ support and mortality, duration and volume of IV fluids, and duration of ICU and hospital stay (Tables 3 and Additional file 1: S3).

### Subgroup analyses

There was no clear difference in the association between mSOFA score and clinical outcomes between patients with and without comorbidities (Additional file 1: Table S4), and in patients who were transferred from other hospitals compared and those admitted directly to HTD (Additional file 1: Table S5).

## Discussion

Here we have presented a modified SOFA score for dengue and shown the utility of both baseline mSOFA and delta mSOFA in predicting clinical outcomes in a dataset of 124 adults with dengue shock admitted to an ED/ICU in Ho Chi Minh City, Vietnam.

In this patient group, although requirement for organ support and mortality were rare events, mSOFA for dengue performed well to discriminate groups by clinical outcomes, including duration of ICU and hospital admission, requirement for organ support and death. Our findings support other studies showing that a one-time SOFA score at either admission, 24 or 48 h can predict mortality in adults with severe dengue [18–24]. Aside from a single study in Brazil, all of these studies were conducted in India, and like the majority of derivation and validation cohorts for the original SOFA score, they all included patients with a higher median age, more comorbidities, higher frequency of organ failure and higher mortality than our own population. Studies from other dengue patient populations, including an elderly cohort in Taiwan, and patients with dengue induced haemophagocytic lymphohistiocytosis in Malaysia, also found that SOFA score is an effective discriminant between survivors and non-survivors [25, 26]. Of note, co-infection was common among these cohorts: 23.4% of patients in the Taiwanese cohort had bacterial and/or fungal bloodstream co-infection [27], and in a cohort of adults with severe dengue admitted to ICU in Brazil, 45% patients received antibiotics, and 22% were treated for septic shock [24]. Co-infection may have had a significant but unmeasurable impact on SOFA score parameters, and might have contributed to the higher mortality reported in these cohorts, compared to our own population.

Dengue management in Vietnam is standardised using guidelines from the Vietnamese Ministry of Health [28]. There may be some variations allowing for flexibility in clinical practice, however initial resuscitation should not vary widely between hospitals. We found the mSOFA and delta mSOFA scores performed similarly in the groups of patients transferred from other hopsital compared to those presenting directly to HTD, and between patients with and without comorbities. This suggests that differences in baseline characteristics may not impact the performance of the scoring system, further indicating its potential for pragmatic application.

Our study adds the following: first application of a modified delta SOFA with the addition of pulse pressure to reflect the unique pathophysiology of dengue, and the first time this score has been reported from Vietnam, where the population is significantly younger than the patient populations in the published literature. We have separately reported the individual component scores which make up the SOFA total, and would recommend this be applied in future studies, in order to improve the understanding and external validity of this composite endpoint [9] and potentially improve future iterations of the scoring system.

We substituted PaO_2_ for SpO_2_ in our modified SOFA score to allow non-invasive assessment of oxygenation. We were unable to evaluate the performance of SpO_2_ relative to PaO_2_, but there is growing consensus that this modification is acceptable where arterial blood gas sampling is either unavailable or contraindicated. Haniffa et al. found that requirements for laboratory parameters as prognostic variables hampered the application of critical care scoring systems in low-to-middle-income countries (LMICs) and suggested that scores using readily available clinical data are preferable [29]. Pandharipande et al. investigated substituting PaO_2_/FiO_2_ ratio for SpO_2_/FiO_2_ ratio, and found that the derived scores predicted outcomes with a similar performance to the original respiratory component [30].

The main modification was the addition of the pulse pressure to the cardiovascular component of the orginial SOFA score. This aimed to capture the unique pathophysiology associated with dengue, whereby a slow vascular leak allows several homeostatic mechanisms to be upregulated, increasing the systemic vascular resistance in response to the gradually worsening hypovolemia [31, 32]. Narrowing of the pulse pressure, characterised by increased diastolic blood pressure with maintained systolic blood pressure, reflects cardiovascular compensation and is not seen in other conditions like septic shock where hypovolaemic shock develops rapidly [2, 33]. Mean blood pressure can overestimate the true perfusion pressure in some diseases with marked increases in systemic venous pressure like dengue [34]. In the WHO management guidelines, pulse pressure is used as a diagnostic and resuscitation target instead of MAP in dengue shock [12, 13]. Hence, our mSOFA score for dengue included narrowed pulse pressure in the cardiovascular score, in addition to mean arterial pressure and vasopressor requirement. The inclusion of pulse pressure in the mSOFA changed the cardiovascular score in 15 patients and did not significantly alter the overall performance of the score. As pulse pressure is an important diagnostic criteria for cardiovascular compromise and therapeutic target, particularly for paediatric dengue shock syndrome, we suggest this now needs further evaluation in cohorts of both adults and children with severe dengue. As pulse pressure is simple and routinely measured in all DSS cases ongoing prospective evaluation will not require extra data collection, add cost, or inconvenience the patients. However, our results show that although pulse pressure was associated with some clinical outcomes, mSOFA was a superior predictor of clinical endpoints in this population with established dengue shock.

Despite the accumulating evidence that the SOFA score, modified or otherwise, is useful in dengue prognostication, there is uncertainty about whether all components of the original score contribute useful information. In particular, the benefit of the coagulation component is unclear. In dengue, thrombocytopaenia is multifactorial in aetiology: likely a combination of bone marrow suppression and peripheral platelet consumption by both immune mechanisms and disseminated intravascular coagulation. Thus, thrombocytopaenia may reflect typical disease pathophysiology, rather than true end organ failure. Indeed, sub-analyses investigating the contribution of individual organ parameters to the prognostic capability of the SOFA score in dengue have indicated that respiratory, cardiovascular and neurological impairment have the greatest association with prognosis, with the coagulation component inflating the SOFA score, but adding little or no benefit in mortality prediction [18, 22, 23, 25, 35]. Although this effect will not limit comparison between patients with dengue, it may misrepresent the severity of organ failure if directly compared with other diseases.

In this study, we evaluated for the first time whether using the dynamic delta modified SOFA score might be a feasible endpoint for future therapeutic trials in dengue. We found that delta mSOFA between baseline and 48 h was associated with requirement for organ support, duration of treatment and mortality. This supports a systematic review showing that delta SOFA, rather than other SOFA derivatives, was most significantly associated with mortality by meta-regression (slope 0.7, 95% CI 0.26:1.14, p = 0.004, I2 = 0%), explaining 32% of overall mortality. By contrast, fixed day SOFA was not significantly associated with mortality [11]. Delta SOFA has demonstrated applicability as an endpoint for both pharmacological [36] and non-pharmacological interventions [37] in critical care randomized controlled trials. We plan to use the modified delta SOFA for dengue as an endpoint for the first time in an upcoming clinical trial evaluating host directed therapeutics in moderate-severe dengue [38].

While this is the first effort to evaluate a modified SOFA score for dengue in adults with dengue shock, our study is not without limitations. First, our data only contains patients with a primary diagnosis of dengue shock; rather than severe dengue which encompasses organ impairment and bleeding, albeit several had co-existent liver or renal impairment. This is both a strength and limitation; allowing us to investigate the utility of the modified SOFA score for dengue in a population with a distinct clinical phenotype, while also limiting generalizability to patients with other types of severe dengue. The lack of bilirubin data at 48 h post enrolment hinders the ability to evaluate the liver derangement using the delta mSOFA score. A larger sample size is needed in order to evaluate uncommon complications in dengue, for example dengue primarily affecting the central nervous system. Going forward, we plan to apply this mSOFA score for dengue prospectively to adult patients with all phenotypes of severe dengue in the ICU using an established electronic data registry.

Secondly, we do not include children in this study. In hyper-endemic regions, children and adolescents represent the majority of severe dengue cases; we cannot simply extrapolate our findings to children because the normal range of clinical observations, presentation and outcomes of severe dengue differ between children and adults. Children present more frequently with pure dengue shock, and adults more frequently with mixed organ impairment [39]. We plan to continue this work by assessing this mSOFA score in children with dengue shock and tailoring it to a specific pediatric score as necessary.

Ultimately, a balance must be struck between the external validity that using a generic score affords, while capturing the unusual pathophysiology of dengue shock, and maximizing applicability to the LMIC setting. Our findings from this study suggest that mSOFA for dengue is associated with meaningful clinical outcomes and could be useful for prognosticating patients admitted with dengue shock. We believe that delta mSOFA for dengue should now be evaluated in action as an endpoint for clinical trials in moderate-severe dengue.

## Supplementary Information

**Additional file 1: Table S1.** Clinical data by patients with and without comorbidities. **Table S2.** Clinical data by patients who were transferred from other hospitals and those admitted to HTD. **Table S3.** Results from models with baseline SOFA and delta SOFA score. All parameters are estimated from models with only one predictor ‘baseline SOFA score’ or ‘delta SOFA score’ with a linear effect. Logistic regression model is used for binary endpoint (ICU admission, mechanical ventilation, vasopressor support, haemofiltration, and mortality), Cox proportional hazard model is used for time-to-event endpoints (ICU discharge, hospital discharge, and stop IV fluid use), and linear regression model is used for total volume of IV fluid use with log-transformation. The estimates (OR, HR, or MR) and 95% CIs are reported for each point increase of baseline SOFA or delta SOFA score. AUC, area under the curve; CI; confidence interval; Est, estimate; HR, hazard ratio; ICU, intensive care unit; IV, intravenous fluid; MR, mean ratio; OR, odds ratio. **Table S4.** Association between baseline mSOFA & delta mSOFA scores and endpoints by patients with and without comorbidities. **Table S5.** Association between baseline mSOFA & delta mSOFA scores and endpoints by patients who were transferred from other hospitals and those admitted to HTD. **Table S6.** Association between Pulse Pressure and narrow Pulse Pressure (Pulse Pressure < 20 mmHg) and endpoints.

## Data Availability

The datasets used during the current study are available from the corresponding author on reasonable request.

## Abbreviations

SOFA: Sequential Organ Failure Assessment
ICU: Intensive care unit
IV: Intravenous
DENV: Dengue virus
ED: Emergency department
WHO: World Health Organisation
OR: Odds ratio
CI: Confidence interval
AUC: Area under the curve
HR: Hazard ratio
HTD: Hospital for tropical diseases
