# Supplementary material for: A modified Sequential Organ Failure Assessment score for dengue: development, evaluation and proposal for use in clinical trials
Source: BMC Infect Dis. 2022 Sep 3;22:722. doi: 10.1186/s12879-022-07705-8 (PMC9441074; doi:10.1186/s12879-022-07705-8)
Supplement: Supplementary file 1 — Additional file 1: Table S1. Clinical data by patients with and without comorbidities. Table S2. Clinical data by patients who were transferred from other hospitals and those admitted to HTD. Table S3. Results from models with baseline SOFA and delta SOFA score. All parameters are estimated from models with only one predictor ‘baseline SOFA score’ or ‘delta SOFA score’ with a linear effect. Logistic regression model is used for binary endpoint (ICU admission, mechanical ventilation, vasopressor support, haemofiltration, and mortality), Cox proportional hazard model is used for time-to-event endpoints (ICU discharge, hospital discharge, and stop IV fluid use), and linear regression model is used for total volume of IV fluid use with log-transformation. The estimates (OR, HR, or MR) and 95% CIs are reported for each point increase of baseline SOFA or delta SOFA score. AUC, area under the curve; CI; confidence interval; Est, estimate; HR, hazard ratio; ICU, intensive care unit; IV, intravenous fluid; MR, mean ratio; OR, odds ratio. Table S4. Association between baseline mSOFA & delta mSOFA scores and endpoints by patients with and without comorbidities. Table S5. Association between baseline mSOFA & delta mSOFA scores and endpoints by patients who were transferred from other hospitals and those admitted to HTD. Table S6. Association between Pulse Pressure and narrow Pulse Pressure (Pulse Pressure < 20 mmHg) and endpoints. [file 12879_2022_7705_MOESM1_ESM.docx]

|  | N | Without comorbidities (N=115) | With comorbidities (N=9) | p-value |
| --- | --- | --- | --- | --- |
| Age (years) | 124 | 24.0 (20.0; 31.0) | 41.0 (29.0; 53.0) | 0.006 |
| Sex male | 124 | 61 (53.0) | 2 (22.2) | 0.093 |
| Transferred from other hospital | 124 | 67 (58.3) | 7 (77.8) | 0.311 |
| Antibiotics in prior hospital | 72 | 1 (1.5) | 0 (0.0) | 1 |
| Intravenous fluid in prior hospital | 74 | 52 (77.6) | 6 (85.7) | 1 |
| Total volume of IV fluid in prior hospital (l) | 56 | 2.0 (1.4; 3.0) | 1.6 (1.5; 3.1) | 0.958 |
| BMI (kg/m2) | 124 | 22.5 (19.7; 26.3) | 24.4 (23.5; 26.8) | 0.182 |
| DOI at shock | 124 | 5.0 (4.0; 5.0) | 5.0 (4.0; 6.0) | 0.925 |
| Admitted to ICU | 124 | 24 (20.9) | 5 (55.6) | 0.032 |
| - Number of days in ICU | 29 | 3.0 (1.8; 5.0) | 3.0 (3.0; 4.0) | 0.579 |
| Total number of days in hospital | 124 | 5.0 (4.0; 7.0) | 5.0 (5.0; 7.0) | 0.787 |
| Required mechanical ventilation | 124 | 6 (5.2) | 3 (33.3) | 0.018 |
| - Number of days required mechanical ventilation | 9 | 7.0 (6.2; 13.8) | 4.0 (3.0; 6.0) | 0.364 |
| Required vasopressor | 124 | 6 (5.2) | 2 (22.2) | 0.105 |
| - Number of days required vasopressor | 8 | 3.0 (1.5; 6.0) | 3.0 (2.5; 3.5) | 1 |
| Number of days required intravenous fluid | 124 | 2.0 (2.0; 2.0) | 2.0 (2.0; 3.0) | 0.403 |
| Total volume of intravenous fluid (l) | 124 | 4.7 (3.6; 6.2) | 4.8 (3.1; 11.9) | 0.821 |
| Received platelet transfusion | 124 | 7 (6.1) | 5 (55.6) | <0.001 |
| Total volume of platelet transfusion (ml) | 12 | 250 (250; 1,250) | 250 (250; 250) | 0.587 |
| Required haemofiltration | 124 | 4 (3.5) | 2 (22.2) | 0.061 |
| - Number of days required haemofiltration | 6 | 7.0 (6.0; 9.2) | 3.0 (2.5; 3.5) | 0.240 |
| Death | 124 | 3 (2.6) | 2 (22.2) | 0.042 |

### Table S1. Clinical data by patients with and without comorbidities

### Table S2. Clinical data by patients who were transferred from other hospitals and those admitted to HTD

|  | N | Admitted to HTD (N=50) | Transferred from other hospital (N=74) | p-value |
| --- | --- | --- | --- | --- |
| Age (years) | 124 | 26.0 (20.0; 32.0) | 24.0 (20.0; 31.8) | 0.628 |
| Sex male | 124 | 23 (46.0) | 40 (54.1) | 0.464 |
| Comorbidities | 124 | 2 (4.0) | 7 (9.5) | 0.311 |
| Antibiotics in prior hospital | 72 | - | 1 (1.4) | - |
| Intravenous fluid in prior hospital | 74 | - | 58 (78.4) | - |
| Total volume of IV fluid in prior hospital (l) | 56 | - | 2.0 (1.5; 3.0) | - |
| BMI (kg/m2) | 124 | 22.3 (20.0; 25.3) | 23.7 (19.8; 27.6) | 0.340 |
| DOI at shock | 124 | 5.0 (4.0; 5.0) | 5.0 (4.0; 5.0) | 0.722 |
| Admitted to ICU | 124 | 9 (18.0) | 20 (27.0) | 0.285 |
| - Number of days in ICU | 29 | 2.0 (1.0; 3.0) | 3.5 (2.0; 5.0) | 0.223 |
| Total number of days in hospital | 124 | 5.0 (4.0; 6.0) | 6.0 (5.0; 7.0) | 0.052 |
| Required mechanical ventilation | 124 | 2 (4.0) | 7 (9.5) | 0.311 |
| - Number of days required mechanical ventilation | 9 | 14.0 (11.0; 17.0) | 6.0 (3.5; 7.0) | 0.106 |
| Required vasopressor | 124 | 1 (2.0) | 7 (9.5) | 0.142 |
| - Number of days required vasopressor | 8 | 3.0 (3.0; 3.0) | 3.0 (1.5; 5.5) | >0.999 |
| Number of days required intravenous fluid | 124 | 2.0 (2.0; 2.0) | 2.0 (2.0; 2.0) | 0.989 |
| Total volume of intravenous fluid (l) | 124 | 5.5 (4.4; 6.6) | 4.2 (2.9; 6.0) | <0.001 |
| Received platelet transfusion | 124 | 2 (4.0) | 10 (13.5) | 0.121 |
| Total volume of platelet transfusion (ml) | 12 | 500 (375; 625) | 250 (250; 475) | 0.719 |
| Required haemofiltration | 124 | 1 (2.0) | 5 (6.8) | 0.400 |
| - Number of days required haemofiltration | 6 | 7.0 (7.0; 7.0) | 4.0 (3.0; 7.0) | 0.766 |
| Death | 124 | 0 (0.0) | 5 (6.8) | 0.081 |

### Table S3. Results from models with baseline SOFA and delta SOFA score

|  |  | Baseline SOFA score | | | | Delta SOFA score | | | |
| --- | --- | --- | --- | --- | --- | --- | --- | --- | --- |
|  | Est | Est (95% CI) | p | Scaled Brier score (95% CI) | AUC (95% CI) | Est (95% CI) | p | Scaled Brier score (95% CI) | AUC (95% CI) |
| ICU admission | OR | 1.55 (1.22; 2.07) | <0.001 | 0.15 (0.03; 0.30) | 0.67 (0.55; 0.79) | 1.73 (1.32; 2.47) | <0.001 | 0.22 (0.06; 0.35) | 0.71 (0.58; 0.81) |
| ICU discharge | HR | 0.60 (0.47; 0.76) | <0.001 | - | - | 0.66 (0.53; 0.82) | <0.001 | - | - |
| Hospital discharge | HR | 0.66 (0.58; 0.75) | <0.001 | - | - | 0.82 (0.75; 0.89) | <0.001 | - | - |
| Stop IV fluid use | HR | 0.82 (0.75; 0.90) | <0.001 | - | - | 0.76 (0.68; 0.83) | <0.001 | - | - |
| Total volume of IV fluid use | MR | 1.18 (1.12; 1.25) | <0.001 | - | - | 1.20 (1.15; 1.25) | <0.001 | - | - |
| Mechanical ventilation | OR | 3.56 (2.05; 7.94) | <0.001 | 0.56 (0.25; 0.89) | 0.91 (0.75; 1.00) | 3.73 (1.99; 9.85) | <0.001 | 0.68 (0.27; 0.96) | 0.93 (0.80; 1.00) |
| Vasopressor support | OR | 3.49 (1.99; 8.00) | <0.001 | 0.51 (0.18; 0.84) | 0.94 (0.83; 1.00) | 2.02 (1.44; 3.34) | <0.001 | 0.64 (0.04; 0.97) | 0.80 (0.51; 1.00) |
| Haemofiltration | OR | 5.81 (2.41; 31.3) | 0.004 | 0.57 (0.26; 0.95) | 0.99 (0.96; 1.00) | 5.74 (2.07; 1278) | <0.001 | 1.00 (0.88; 1.00) | 1.00 (1.00; 1.00) |
| Mortality | OR | 5.49 (2.23; 35.5) | 0.009 | 0.74 (0.22; 0.97) | 0.99 (0.95; 1.00) | 3.59 (1.79; 147) | <0.001 | 1.00 (0.84; 1.00) | 1.00 (1.00; 1.00) |

All parameters are estimated from models with only one predictor ‘baseline SOFA score’ or ‘delta SOFA score’ with a linear effect. Logistic regression model is used for binary endpoint (ICU admission, mechanical ventilation, vasopressor support, haemofiltration, and mortality), Cox proportional hazard model is used for time-to-event endpoints (ICU discharge, hospital discharge, and stop IV fluid use), and linear regression model is used for total volume of IV fluid use with log-transformation. The estimates (OR, HR, or MR) and 95% CIs are reported for each point increase of baseline SOFA or delta SOFA score. AUC, area under the curve; CI; confidence interval; Est, estimate; HR, hazard ratio; ICU, intensive care unit; IV, intravenous fluid; MR, mean ratio; OR, odds ratio.

### Table S4. Association between baseline mSOFA & delta mSOFA scores and endpoints by patients with and without comorbidities

|  |  | Baseline mSOFA score | | | | Delta mSOFA score | | | |
| --- | --- | --- | --- | --- | --- | --- | --- | --- | --- |
|  |  | Without comorbidities | | With comorbidites | | Without comorbidities | | With comorbidites | |
| Outcome | Est | Est (95% CI) | p | Est (95% CI) | p | Est (95% CI) | p | Est (95% CI) | p |
| ICU admission | OR | 1.55 (1.17; 2.12) | 0.004 | 2.78 (1.09; 35.02) | 0.186 | 1.55 (1.20; 2.19) | 0.004 | 1.48 (0.85; 3.82) | 0.260 |
| ICU discharge | HR | 0.61 (0.47; 0.78) | <0.001 |  |  | 0.70 (0.56; 0.87) | 0.001 | 0.48 (0.14; 1.61) | 0.233 |
| Hospital discharge | HR | 0.66 (0.58; 0.75) | <0.001 | 0.53 (0.31; 0.91) | 0.021 | 0.84 (0.77; 0.92) | <0.001 | 0.76 (0.55; 1.06) | 0.105 |
| Stop IV fluid use | HR | 0.84 (0.75; 0.93) | <0.001 | 0.73 (0.53; 1.01) | 0.059 | 0.78 (0.70; 0.86) | <0.001 | 0.56 (0.33; 0.94) | 0.029 |
| Total volume of fluid use | MR | 1.17 (1.10; 1.24) | <0.001 | 1.30 (1.02; 1.66) | 0.040 | 1.17 (1.11; 1.23) | <0.001 | 1.37 (1.24; 1.52) | <0.001 |
| Mechanical ventilation | OR | 3.80 (2.04; 9.53) | <0.001 | 2.74 (1.15; 68.56) | 0.194 | 3.05 (1.68; 8.51) | 0.008 |  |  |
| Vasopressor support | OR | 3.80 (2.04; 9.53) | <0.001 |  |  | 1.80 (1.30; 2.87) | 0.002 |  |  |
| Haemofiltration | OR | 5.77 (2.34; 33.75) | 0.005 |  |  | 3.50 (1.70; 112.88) | <0.001 | 1.86 (0.91; 10.49) | 0.099 |
| Mortality | OR | 4.22 (1.87; 19.83) | 0.007 |  |  | 2.82 (1.66; 14.75) | <0.001 | 1.86 (0.91; 10.49) | 0.099 |

**Table S5. Association between baseline mSOFA & delta mSOFA scores and endpoints by patients who were transferred from other hospitals and those admitted to HTD**

|  |  | Baseline mSOFA score | | | | Delta mSOFA score | | | |
| --- | --- | --- | --- | --- | --- | --- | --- | --- | --- |
|  |  | Transferred from other hospital | | Admitted to HTD | | Transferred from other hospital | | Admitted to HTD | |
| Outcome | Est | Est (95% CI) | p | Est (95% CI) | p | Est (95% CI) | p | Est (95% CI) | p |
| ICU admission | OR | 1.64 (1.23; 2.35) | 0.003 | 1.70 (1.05; 3.02) | 0.043 | 2.11 (1.39; 3.85) | 0.005 | 1.05 (0.66; 1.70) | 0.838 |
| ICU discharge | HR | 0.66 (0.52; 0.84) | <0.001 | 0.68 (0.46; 1.00) | 0.052 | 0.69 (0.54; 0.89) | 0.004 | 0.81 (0.58; 1.12) | 0.202 |
| Hospital discharge | HR | 0.66 (0.57; 0.77) | <0.001 | 0.68 (0.56; 0.83) | <0.001 | 0.81 (0.72; 0.90) | <0.001 | 0.92 (0.77; 1.09) | 0.332 |
| Stop IV fluid use | HR | 0.78 (0.69; 0.88) | <0.001 | 0.91 (0.78; 1.06) | 0.204 | 0.76 (0.67; 0.86) | <0.001 | 0.79 (0.67; 0.93) | 0.005 |
| Total volume of fluid use | MR | 1.23 (1.14; 1.32) | <0.001 | 1.15 (1.08; 1.22) | <0.001 | 1.23 (1.16; 1.30) | <0.001 | 1.07 (1.00; 1.15) | 0.052 |
| Mechanical ventilation | OR | 3.01 (1.74; 7.18) | 0.002 |  |  | 3.39 (1.69; 20.41) | 0.032 | 5.80 (1.53; 87.71) | 0.048 |
| Vasopressor support | OR | 3.01 (1.74; 7.18) | 0.002 |  |  | 1.78 (1.29; 2.88) | 0.003 |  |  |
| Haemofiltration | OR | 4.65 (2.01; 27.87) | 0.012 |  |  | 3.09 (1.69; 48.99) | <0.001 | 3.75 (0.98; 100.43) | 0.053 |
| Mortality | OR | 4.65 (2.01; 27.87) | 0.012 |  |  | 3.09 (1.69; 48.99) | <0.001 |  |  |

### Supplementary Table 6. Association between Pulse Pressure and narrow Pulse Pressure (Pulse Pressure < 20 mmHg) and endpoints

|  |  | Pulse pressure (mmHg) | | | | Narrow pulse pressure (pulse pressure < 20 mmHg) | | | |
| --- | --- | --- | --- | --- | --- | --- | --- | --- | --- |
|  | Est | Est (95% CI) | p | Scaled Brier score (95% CI) | AUC (95% CI) | Est (95% CI) | p | Scaled Brier score (95% CI) | AUC (95% CI) |
| ICU admission | OR | 0.90 (0.83; 0.95) | 0.001 | 0.11 (0.02; 0.30) | 0.72 (0.61; 0.82) | 9.47 (3.02; 33.48) | <0.001 | 0.16 (0.03; 0.33) | 0.65 (0.57; 0.74) |
| ICU discharge | HR | 0.98 (0.94; 1.03) | 0.482 |  |  | 1.00 (0.46; 2.18) | 0.993 |  |  |
| Hospital discharge | HR | 1.02 (1.00; 1.04) | 0.033 |  |  | 0.48 (0.28; 0.82) | 0.008 |  |  |
| Stop IV fluid use | HR | 1.01 (0.99; 1.04) | 0.175 |  |  | 0.62 (0.36; 1.07) | 0.089 |  |  |
| Total volume of IV fluid use | MR | 0.99 (0.97; 1.00) | 0.103 |  |  | 1.65 (1.16; 2.34) | 0.005 |  |  |
| Mechanical ventilation | OR | 0.93 (0.84; 1.01) | 0.106 | -0.04 (-0.09; 0.15) | 0.65 (0.50; 0.84) | 4.29 (0.82; 18.67) | 0.059 | -0.04 (-0.11; 0.18) | 0.61 (0.50; 0.78) |
| Vasopressor support | OR | 0.96 (0.87; 1.05) | 0.393 | 0.01 (-0.01; 0.07) | 0.58 (0.47; 0.81) | 5.20 (0.97; 24.08) | 0.037 | 0.01 (-0.01; 0.20) | 0.64 (0.50; 0.82) |
| Haemofiltration | OR | 0.96 (0.86; 1.06) | 0.432 | -0.09 (-0.19; 0.11) | 0.58 (0.45; 0.87) | 4.04 (0.52; 22.93) | 0.127 | -0.09 | 0.61 |
| Mortality | OR | 1.00 (0.89; 1.11) | 0.957 | -0.03 (-0.07; 0.06) | 0.49 (0.42; 0.84) | 1.87 (0.09; 13.85) | 0.586 | -0.03 | 0.54 |
